# Virtual design of novel *Plasmodium falciparum* cysteine protease falcipain-2 hybrid lactone–chalcone and isatin–chalcone inhibitors probing the S2 active site pocket

**DOI:** 10.1080/14756366.2018.1564288

**Published:** 2019-01-30

**Authors:** Koffi N’Guessan Placide Gabin Allangba, Mélalie Keita, Raymond Kre N’Guessan, Eugene Megnassan, Vladimir Frecer, Stanislav Miertus

**Affiliations:** a Laboratoire de Physique Fondamentale et Appliquée (LPFA), University of Abobo Adjamé (now Nangui Abrogoua), Abidjan, Côte d’Ivoire;; b Laboratoire de Chimie Organique Structurale et Théorique, University of Cocody (now Felix Houphouët Boigny), Abidjan, Côte d'Ivoire;; c ICS-UNIDO, Trieste, Italy;; d Faculty of Pharmacy, Comenius University in Bratislava, Bratislava, Slovakia;; e International Centre for Applied Research and Sustainable Technology, Bratislava, Slovakia;; f Faculty of Natural Sciences, University of SS. Cyril and Methodius, Trnava, Slovakia

**Keywords:** Falcipain-2, Plasmodium falciparum, molecular modelling, QSAR model, pharmacophore, virtual library, pharmacokinetics

## Abstract

We report computer-aided design of new lactone–chalcone and isatin–chalcone (HLCIC) inhibitors of the falcipain-2 (*Pf*FP-2). 3D models of 15 FP-2:HLCIC1-15 complexes with known observed activity (*IC*_50_^exp^) were prepared to establish a quantitative structure–activity (QSAR) model and linear correlation between relative Gibbs free energy of enzyme:inhibitor complex formation (ΔΔ*G*_com_) and *IC*_50_^exp^: p*IC*_50_^exp^ = −0.0236 × ΔΔ*G*_com_+5.082(#); *R* ^2^ = 0.93. A 3D pharmacophore model (PH4) derived from the QSAR directed our effort to design novel HLCIC analogues. During the design, an initial virtual library of 2621440 HLCIC was focused down to 18288 drug-like compounds and finally, PH4 screened to identify 81 promising compounds. Thirty-three others were added from an intuitive substitution approach intended to fill better the enzyme S2 pocket. One hundred and fourteen theoretical IC_50_ (*IC*_50_^pre)^ values were predicted by means of (#) and their pharmacokinetics (ADME) profiles. More than 30 putative HLCICs display *IC*_50_^pre^ 100 times superior to that of the published most active training set inhibitor HLCIC1.

## 1. Introduction

Malaria remains the main cause of death all over the world and we are unable to reverse this sad morbidity statistic. Indeed, during the year 2015, 212 million cases of malaria and 429 thousand death cases were reported by the WHO^1^. *Plasmodium falciparum* (*Pf*), the most virulent causative agent of malaria, is developing resistance rapidly so that the future of the Artemisinin Combined Therapy (ACT, available since 2006) is compromised^2^. For almost a decade the development of novel antimalarials concerned mainly ACT, as recently exemplified by introduction of the combination of artemether and lumefantrine referred to as *novamether* ^3^. Luckily, this alarming dark picture has been illuminated by the reported new antimalarial candidates^4–6^. Relatively low level of structural knowledge of novel and validated pharmacological targets of the *Pf* on one side and the lack of inhibition pharmacophores on the other, remain the drawbacks of antimalarial drug design (ADD) and development. One of the recent ADD strategies was to design hybrids molecules containing artemisinin (ART) or other antimalarial combined with another structural fragment^6–8^. Falcipains have drawn the attention of ADD due to their important role in the haemoglobin degradation (along with plasmepsins) while their inhibition is lethal to the *Pf* ^9–11^. Lactone–chalcone and isatin–chalcone (HLCIC) hybrid molecules have been mentioned in this context, their experimental FP-2 inhibitory activities were reported^12^. They essentially target the bipartite motifs of falcipain-2 (FP-2) and falcipain-3 (FP-3), the papain-family cysteine proteases of *Pf*, which are involved in the degradation of host’s haemoglobin within the food vacuole during the blood-stage of the parasite^13^ ^,^ ^14^. Due to their specific features, falcipains represent promising targets for the development of next-generation antimalarials^10^ ^,^ ^14^.

**Figure 1.**
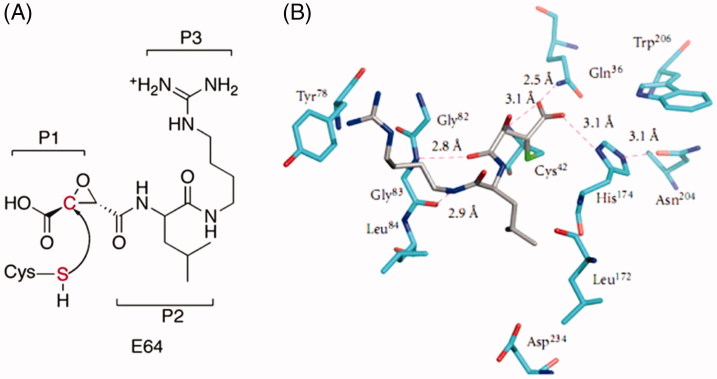
(a) Molecular structure of epoxysuccinate E64 with indicated P1, P2, and P3 positions. (b) 3D depiction of E64 interactions with the FP-2 active site residues involving residues that occupy S1, S2, and S3 pockets of the active site^15^.

In this work, we design new analogues of HLCIC starting from a series of 15 known HLCIC hybrids with determined experimental inhibition potencies (*IC*_50_^exp^), which were used as a training set^12^. We build on our design and new analogue activity prediction relies on a reliable descriptor, namely standard Gibbs free energy (GFE) of enzyme:inhibitor (E:I) complex formation (Δ*G*_com_), quantitative structure–activity relationships (QSAR), 3D structural model of the FP-2 and analysis of the inhibitor–enzyme interactions. Relative changes in the GFE of E:I complex formation were computed in order to build a linear regression QSAR model utilising the published *IC*_50_^exp^ ^12^. Each complex was carefully built by *in situ* modification of the reference crystal structure of FP-2 (3BPF)^15^ in complex with epoxysuccinate E64 (Figure 1). The 3D models of inhibitors bound to FP-2, QSAR and pharmacophore (PH4) models derived for the training set compounds provided the necessary structural information needed to improve inhibitor interactions at pockets S1, S2, and S3 of the FP-2 active site. Screening of designed virtual library (VL) of analogues by the PH4 led to the identification of potent HLCIC, which are predicted to be hundreds of times more potent than the best training set inhibitor HLCIC1 (*IC*_50_^exp^ = 6.8 µM). 

## 2. Materials and methods

**Figure 2.**
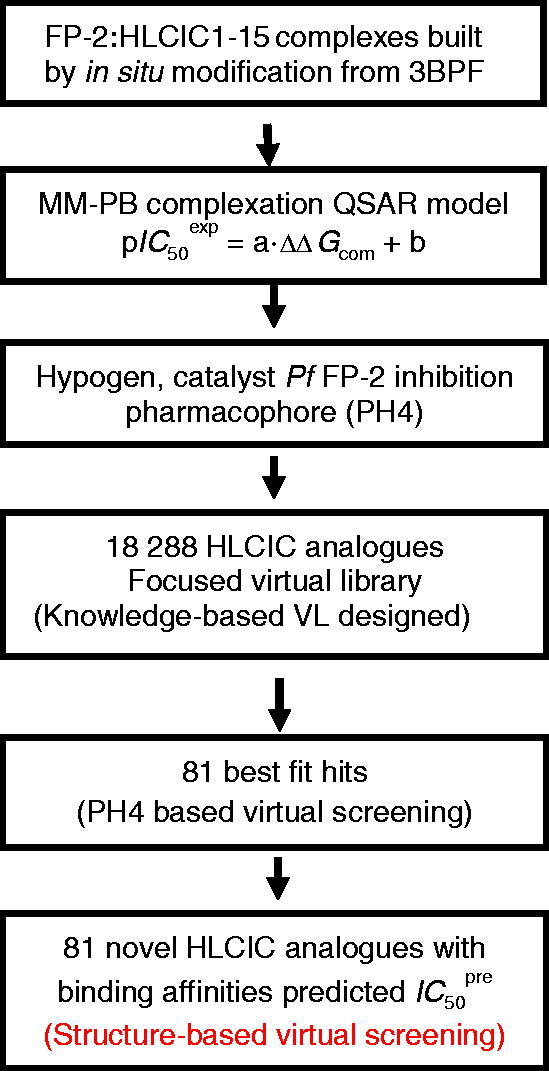
Workflow describing the multistep approach to virtually designed novel HLCIC analogues with higher predicted potencies against the FP-2 of *Pf*.

The methodology of computer-assisted molecular design based on 3D models of E:I complexes and QSAR analysis of a training set of known inhibitors has been successfully applied to optimisation of antiviral, antibacterial, and anti-protozoan compounds including peptidomimetic, hydroxynaphthoic, thymidine, triclosan, pyrrolidine carboxamide derivatives, peptidic, and ART hybrids inhibitors^8^ ^,^ ^16–26^. The workflow shown in Figure 2 describes the series of steps of virtual design of novel HLCIC analogues.

### 2.1. Training sets

The chemical structures of the training set compounds comprising 15 HLCIC hybrids and their experimental biological activities (IC_50_^exp^ = 6.8–90 µM) are taken from the literature^12^. The IC_50_^exp^ values of the HLCIC compounds cover a relatively wide concentration range, which is needed to build a reliable QSAR model.

### 2.2. Model building and calculation of binding affinity

Molecular modelling was carried out for the E:I (FP-2:HLCIC) complexes, the free enzyme FP-2, and the free HLCIC inhibitors starting from the high-resolution crystal structure of FP-2 co-crystallised with epoxysuccinate E64 inhibitor (*PDB* code 3BPF, resolution 2.9 Å) using Insight II molecular modelling program^27^. Initially, all crystallographic waters were removed, then hydrogens were added to the residues of the FP-2 and FP-2:HLCIC complex with the protonisation/ionisation state corresponding to the pH of 7 keeping the N- and C-terminal groups neutral. Inhibitors were modelled from the 3BPF reference crystal structure by *in situ* modification of functional groups in the molecular scaffold of the endogenous E64 inhibitor. All rotatable bonds of the replacing fragments were subjected to an exhaustive conformational search coupled with a careful gradual energy-minimisation of the modified inhibitor and active-site residues of FP-2 located in the immediate vicinity (5 Å radius) in order to identify low-energy bound conformations of the modified inhibitors. The resulting low-energy structures of the E:I complexes were then carefully refined by energy-minimisation procedure of the entire complex to obtain stable structures of the binary FP-2:HLCIC complexes. The complete description of the computation of relative ligand binding affinity (ΔΔ*G*_com_) is described in Ref. [25]:

(1) $$\Delta \Delta G_{\text{com}}=\Delta G_{\text{com}}\left(I\right)-\Delta G_{\text{com}}\left(I_{\text{ref}}\right)=\Delta \Delta H_{\text{MM}}-\Delta \Delta TS_{\text{vib}}+\Delta \Delta G_{\text{sol}}$$

The ΔΔ*H*_MM_ describes the relative enthalpic contribution to the GFE change corresponding to the intermolecular interactions in the E:I complex estimated by molecular mechanics (MM), ΔΔ*G*_sol_, and ΔΔ*TS*_vib_ represent the relative solvation and vibrational entropy contributions to the GFE of the E:I complex formation, respectively.

### 2.3. Molecular mechanics

Modelling of the inhibitors and their complexes were carried out in all-atom representation using atomic parameters and charges of the class II consistent force field CFF91^22^. A dielectric constant of 4 was used for all MM calculations in order to take into account the dielectric shielding effect in proteins. Minimisations of the E:I complexes, free E and I were carried out by relaxing the structures gradually, starting with added hydrogen atoms, continued with residue side chain heavy atoms and followed by the protein backbone relaxation. Geometry optimisations were performed using the sufficient number of steepest descent and conjugate gradient iterative cycles and average gradient convergence criterion of 0.01 kcal·mol^−1^·Å^−1^.

### 2.4. Solvation GFE

The electrostatic component of the solvation GFE, which includes also the effect of ionic strength of the solvent by solving the non-linear Poisson–Boltzmann equation^28^ ^,^ ^29^ was computed by the DelPhi module of the Discovery Studio (DS 2.5)^30^. The program represents the solvent by a continuous medium of high dielectric constant (*ε*_ro_ = 80) and the solute as a charge distribution filling a low dielectric cavity (*ε*_ri_ = 4) with boundaries linked to the solute’s molecular surface. The program numerically solves for the molecular electrostatic potential and reaction field around the solute using finite difference method. DelPhi calculations were done on a (235 × 235 × 235) cubic lattice grid for the E:I complexes and free E and on a (65 × 65 × 65) grid for the free I. Full coulombic boundary conditions were employed. Two subsequent focusing steps led to a similar final resolution of about 0.3 Å per grid unit at 70% filling of the grid by the solute. Physiological ionic strength of 0.145 mol·dm^−3^, atomic partial charges and radii defined in the CFF force field parameter set^30^ and a probe sphere radius of 1.4 Å were used. The electrostatic component of the Poisson–Boltzmann solvation GFE was calculated as the reaction field energy^21^ ^,^ ^24^ ^,^ ^29^ ^,^ ^31^ ^,^ ^32^.

### 2.5. Interaction energy

The molecular mechanic interaction energy (*E*_int_) calculation protocol available in DS 2.5^30^ was used to compute the non-bonded interactions (van der Walls and electrostatic interatomic potential terms) between two sets of atoms belonging either to the E or I in the E:I complexes. All pairs of interactions of the total enzyme–inhibitor interaction energy were evaluated using CFF force field parameters with a relative permittivity of 4^30^. In particular, the breakdown of *E*_int_ into contributions from individual active site residues allows a quantitative analysis, which permits identification of residues with the highest contribution to the ligand binding. It also helps with the identification of favourable structural modifications and suggests molecular moieties in the inhibitor structure which are primarily responsible for the biological activity of the compound^22^.

### 2.6. 3D-QSAR pharmacophore generation

Pharmacophore (PH4) modelling assumes that a set of key structural features responsible for biological activity of the compound is recognised by the active site during receptor binding. In this work, the pharmacophore was prepared by the 3D-QSAR pharmacophore protocol of Catalyst HypoGen algorithm^33^ implemented in DS 2.5^30^. Bound conformations of HLCIC inhibitors taken from the refined E:I complexes were considered for construction of the PH4. The top scoring pharmacophore hypothesis was prepared in three stages: constructive, subtractive, and optimisation step, from a set of most active HLCIC inhibitors. The inactive compounds served for the definition of excluded volume. During the PH4 generation, five features available in the HypoGen algorithm were used: hydrophobic aromatic (HYdAr), hydrophobic aliphatic (HYd), hydrogen-bond donor (HBD), acceptor (HBA), and ring aromatic (Ar) feature. Default values of the adjustable parameters were kept during the PH4 generation, except the uncertainty on the biological activity, which was reduced to 1.25 instead of 3. This adjustment modified the uncertainty interval of experimental activity from a wide span [*IC*_50_^exp^/3, 3×*IC*_50_^exp^] to a relatively narrow one] [4×*IC*_50_^exp^/5, 5×*IC*_50_^exp^/4], due to accuracy and homogeneity of the measured activities originating from the same laboratory^12^. The top ten pharmacophores were generated with the number of missing features set to zero. Finally, the best PH4 model was selected. Generally, a PH4 model, as the one described here, can be used to estimate the p*IC*_50_^pre^ of new analogues on the basis of their mapping to its features. In this study, priority was given to PH4 based screening of ADME focused VL of HLCIC analogues.

### 2.7. ADME-related properties

Properties that determine the pharmacokinetics profile of a compound, besides octanol/water partitioning coefficient, aqueous solubility, blood/brain partition coefficient, Caco-2 cell permeability, serum protein binding, number of likely metabolic reactions and other 18 descriptors related to adsorption, distribution, metabolism and excretion (ADME properties) of the inhibitors were computed by the QikProp program^34^ based on the methods of Jorgensen^35–37^. According to these methods, experimental results of more than 710 compounds including about 500 drugs and related heterocycles, were correlated with computed physico-chemical descriptors, resulting in an accurate prediction of molecule’s pharmacokinetic profile. Drug likeness (#stars) is represented by the number of descriptors that exceed the range of values determined for 95% of known drugs out of 24 selected descriptors computed by the QikProp^34^. Drug-likeness was used as the global compound selection criterion related to ADME properties. The selected ADME descriptors were calculated from 3D structures of compounds considered. They were used to assess the pharmacokinetics profile of designed compounds and served also for the VL focusing.

### 2.8. Virtual combinatory library generation

The analogue model building was performed with Molecular Operating Environment (MOE) program^38^. The library of analogues was enumerated by attaching R-groups (fragments, building blocks) onto HLCIC scaffold using the Quasar CombiDesign module of MOE^38^. Reagents and chemicals considered in this paper were selected from the directories of chemicals available from the commercial sources. Each analogue was built as a neutral molecule in the MOE program^38^, its geometry was refined by MM optimisation through smart minimiser of DS 2.5^30^ meeting high convergence criteria (threshold on energy difference of 10^−4 ^kcal·mol^−1^ and root mean square deviation (RMSD) of 10^−5 ^Å), dielectric constant of 4, using class II consistent force field CFF^39^.

### 2.9. ADME-based library focusing

Twenty-four pharmacokinetics-related molecular descriptors available in QikProp^34^, which characterise a wide spectrum of molecular properties as described in Section 2.7, were used. Optimum ranges of these 24 descriptors were defined in terms of upper and lower bounds according to QikProp^34^. Among them predicted drug-likeness (#stars, Section 2.7) was used to retain drug-like HLCIC analogues in the focused VL.

### 2.10. Pharmacophore-based library focusing

The pharmacophore model (PH4) described in Section 2.6 was derived from the bound conformations of HLCIC at the active site of FP-2. The enumerated and ADME-focused VL was further evaluated by using the pharmacophore mapping protocol available of DS 2.5^30^. Within this protocol, each generated conformer of the analogues was geometry optimised by means of the CFF force field for a maximum of 500 energy minimisation steps and subsequently aligned and mapped to the PH4 model in order to select the top ranking overlaps. Twenty best-fitting inhibitor conformers were saved and clustered into 10 conformational families according to their mutual RMSD by Jarvis-Patrick complete linkage clustering method^40^. The best representative of each cluster was considered for the virtual screening of analogues. Only those analogues mapping to all PH4 features were retained for the *in silico* screening.

### 2.11. In silico screening

The conformer with the best match to the PH4 pharmacophore in each cluster of the focused library subset was selected for *in silico* screening by the complexation QSAR model. The relative GFE of E:I complex formation in water ΔΔ*G*_com_ was computed for each selected new analogue and then used for prediction of FP-2 inhibitory potencies (*IC*_50_^pre^) of the focused VL of HLCIC analogues by inserting this parameter into the target-specific scoring function. The scoring function, which is specific for the FP-2 receptor of *Pf* inhibition, is given in Equation (2), was parameterised using the QSAR model of training set of HLCIC inhibitors^12^.

(2) $$pIC_{50}{}^{\text{pre}}=-{\;\text{log}\;}_{10}IC_{50}{}^{\text{pre}}=a\cdot \Delta \Delta G_{\text{com}}+b$$

## 3. Results and discussion

A series of 18 [15 training set HLCIC and 3 validation set (VS) HLCIV] of HLCIC inhibitors and their experimental activities (*IC*_50_^exp^) from the same laboratory^12^ were selected (Table 1). These activities cover a relatively wide range 6.8 µM ≤ *IC*_50_^exp^ ≤ 90 µM and allowed building of a valid QSAR model.

**Table 1.** Training set (HLCIC) and validation set (HLCIV) of FP-2 inhibitors^12^ used in the preparation of QSAR model of inhibitor binding to the FP-2 of *Pf*.

Chalcone scaffold							
linker-isatin (-lki)				linker-lactone (-lkl)			
Training set	*R*_1_	*R*_2_	*R*_3_	*R*_4_	*R*_5_	*R*_6_	*IC*_50_^exp^(μM)
HLCIC1	–OMe	–H	–OMe	H	lki	H	6.80
HLCIC2	–H	–H	–OMe	H	lki	H	10.29
HLCIC3	–OMe	–OMe	–OMe	H	lki	H	11.49
HLCIC4	–H	–H	–OMe	lki	H	H	25.44
HLCIC5	–OMe	–H	–OMe	lki	H	H	15.58
HLCIC6	–OMe	–OMe	–OMe	lki	H	H	90.47
HLCIC7	–H	–H	–OMe	H	H	lki	10.84
HLCIC8	–OMe	–OMe	–OMe	H	H	lkl	28.50
HLCIC9	–H	–H	–lki	H	H	–OMe	15.89
HLCIC10	lki	–H	H	H	H	–OMe	15.96
HLCIC11	lki	–H	H	–OMe	–H	–OMe	15.04
HLCIC12	lki	H	H	–OMe	–OMe	–OMe	15.33
HLCIC13	–H	lki	H	–H	–H	–OMe	9.91
HLCIC14	–H	lki	H	–OMe	–H	–OMe	10.61
HLCIC15	–H	lki	H	–H	–H	–OMe	16.73
Validation set	*R*_1_	*R*_2_	*R*_3_	*R*_4_	*R*_5_	*R*_6_	*IC*_50_^exp^(μM)
HLCICV1	–OMe	–OMe	–OMe	H	H	–lki	16.62
HLCICV2	–H	–H	–lki	–OMe	–OMe	–OMe	25.06
HLCICV3	–H	–H	–lki	–OMe	–H	–OMe	18.28

### 3.1. Results

**Figure 3.**
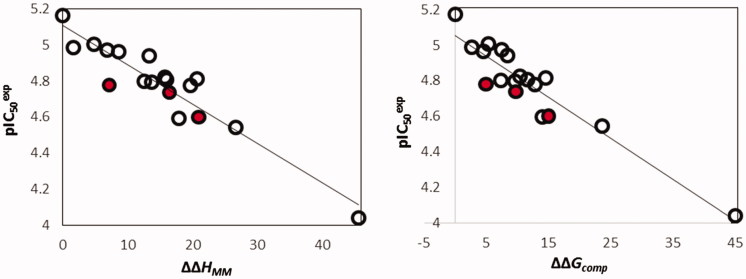
(left): Plot of correlation between p*IC*_50_^exp^ and relative enthalpic contribution to the GFE ΔΔ*H*_MM_; (right) similar plot for relative complexation GFE ΔΔ*G*_com_ of the training set of HLCIC, all in kcal·mol^−1^. Validation set data is shown in red colour.

The relative GFE of E:I complex formation ΔΔ*G*_com_ was calculated for the FP-2: HLCIC complexes as described in Section 2. Table 2 shows GFE and their components (Equation (1)). The ΔΔ*G*_com_ reflects the mutual affinity between the enzyme and the inhibitor. Since it is calculated via an approximate approach, the relevance of the binding model is evaluated by a linear regression with experimentally observed activity data (IC_50_^exp^)^12^ (Equation (2)), which led to a linear correlation and QSAR model for the training set of HLCIC inhibitors. Two correlation equations obtained for the GFE of E:I complex formation ΔΔ*G*_com_ (Equation (B)) and its enthalpic component ΔΔ*H*_MM_ (Equation (A)), respectively are presented in Table 3 with the relevant statistical data plotted in Figure 3. The relatively high values of the regression coefficient *R* ^2^ and the Fischer *F*-test of the correlation involving ΔΔ*G*_com_ indicate that there is a strong relationship between the binding model and the experimental inhibitory potencies of the HLCIC.

**Table 2.** GFE of E:I complex formation (ΔΔ*G*_com_ binding affinity) and its components for the training set of FP-2 inhibitors HLCIC1-15 and the validation set of inhibitors HLCIV1-3.

Training set^a^	*M_w_*^b^	ΔΔ*H*_MM_^c^	ΔΔ*G*_sol_^d^	ΔΔ*TS*_vib_^e^	ΔΔ*G*_com_^f^	*IC*_50_^exp^ ^g^
HLCIC1	607	0	0	0	0	6.80
HLCIC2	577	1.66	−0.39	−1.37	2.64	10.29
HLCIC3	637	13.38	−4.50	0.41	8.47	11.49
HLCIC4	577	17.93	−3.63	0.24	14.06	25.44
HLCIC5	607	15.96	−2.93	1.45	11.57	15.58
HLCIC6	637	45.68	−3.75	−2.99	44.92	90.47
HLCIC7	577	8.64	−3.59	0.45	4.60	10.84
HLCIC8	570	26.70	5.21	8.22	23.69	28.5
HLCIC9	577	12.61	−5.21	0.01	7.39	15.89
HLCIC10	577	13.77	−6.83	−2.70	9.64	15.96
HLCIC11	607	15.73	−5.84	−0.53	10.43	15.04
HLCIC12	637	20.75	−5.80	0.41	14.54	15.33
HLCIC13	577	4.80	−2.49	−3.03	5.34	9.91
HLCIC14	607	6.81	−1.14	−1.87	7.55	10.61
HLCIC15	637	19.63	−4.51	2.29	12.83	16.73
Validation set	*M_w_*^b^	ΔΔ*H*_MM_^c^	ΔΔ*G*_sol_^d^	ΔΔ*TS*_vib_^e^	ΔΔ*G*_com_^f^	p*IC*_50_^pre^/p*IC*_50_^exp^ ^h^
HLCIV1	637	7.13	0.29	2.51	4.91	1.104
HLCIV2	637	20.98	−4.83	1.14	15.01	1.079
HLCIV3	607	16.47	−5.97	0.71	9.78	1.065

^a^For the chemical structures of the HLCIC see Table 1.

^b^ *M_w_* is the molecular mass of the inhibitor (g·mol^−1^).

^c^ΔΔ*H*_MM_ (kcal·mol^−1^) is the relative enthalpic contribution to the GFE change of the E:I complex formation derived by MM: ΔΔ*H*_MM_ ≅ [*E*_tot_{FP-2:HLCICx} − *E*_tot_{HLCICx}] − [*E*_tot_{FP-2:HLCIC1} − *E*_tot_{HLCIC1}] where *E*_tot_ is the MM total energy and HLCIC1 is the reference inhibitor.

^d^ΔΔ*G*_sol_ (kcal·mol^−1^) is the relative solvation contribution to GFE change of the E:I complex formation ΔΔ*G*_sol_ = [*G*_sol_{FP-2:HLCICx} − *G*_sol_{HLCICx}] − [*G*_sol_{FP-2:HLCIC1} − *G*_sol_ {HLCIC1}].

^e^ΔΔ*TS*_vib_ (kcal·mol^−1^) is the relative entropic contribution of the inhibitor to the GFE related to protease-inhibitor complex formation: ΔΔ*TS*_vib_ = [*TS*_vib_{FP-2:HLCICx} − *TS*_vib_ {HLCICx}] − [*TS*_vib_ {FP-2:HLCIC1} − *TS*_vib_ {HLCIC1}].

^f^ΔΔ*G*_com_ (kcal·mol^−1^) is the relative GFE change of E:I complex formation: ΔΔ*G*_com_ ≅ ΔΔ*H*_MM_ + ΔΔ*G*_sol_ − ΔΔ*TS*_vib_.

^g^ *IC*_50_^exp^ (μM) is the experimental half-maximal inhibitory concentration obtained from Ref. [12].

^h^Ratio of predicted and experimental half-maximal inhibition concentrations p*IC*_50_^pre^/p*IC*_50_^exp^ (where p*IC*_50_^pre^ = −log_10_ *IC*_50_^pre^ was predicted from computed ΔΔ*G*_com_ using the regression equation (B) shown in Table 3.

**Table 3.** Regression analysis of computed binding affinities ΔΔ*G*_com_, its enthalpic component ΔΔ*H*_MM_ and observed activity p*IC*_50_^exp^ = −log_10_ *IC*_50_^exp^ ^12^ of hybrid lactone–chalcone and isatin–chalcone HLCIC.

Statistical data of linear regression	(A)	(B)
$pIC_{50}^{\text{exp}\;}=-0.0224\cdot \Delta \Delta H_{\text{MM}}+5.1372$ (A)	–	–
$pIC_{50}^{\text{exp}\;}=-0.0236\cdot \Delta \Delta G_{\text{com}}+5.082$ (B)	–	–
Number of compounds n	15	15
Squared correlation coefficient of regression *R*^2^	0.91	0.93
LOO cross-validated squared correlation coef. *R*_xv_^2^	0.90	0.92
Standard error of regression *σ*	0.08	0.07
Statistical significance of regression, Fischer *F*-test	139.1	180.4
Level of statistical significance *α*	>95%	>95%
Range of activities *IC*_50_^exp^ (µM)	6.8–90.0	

**Figure 4.**
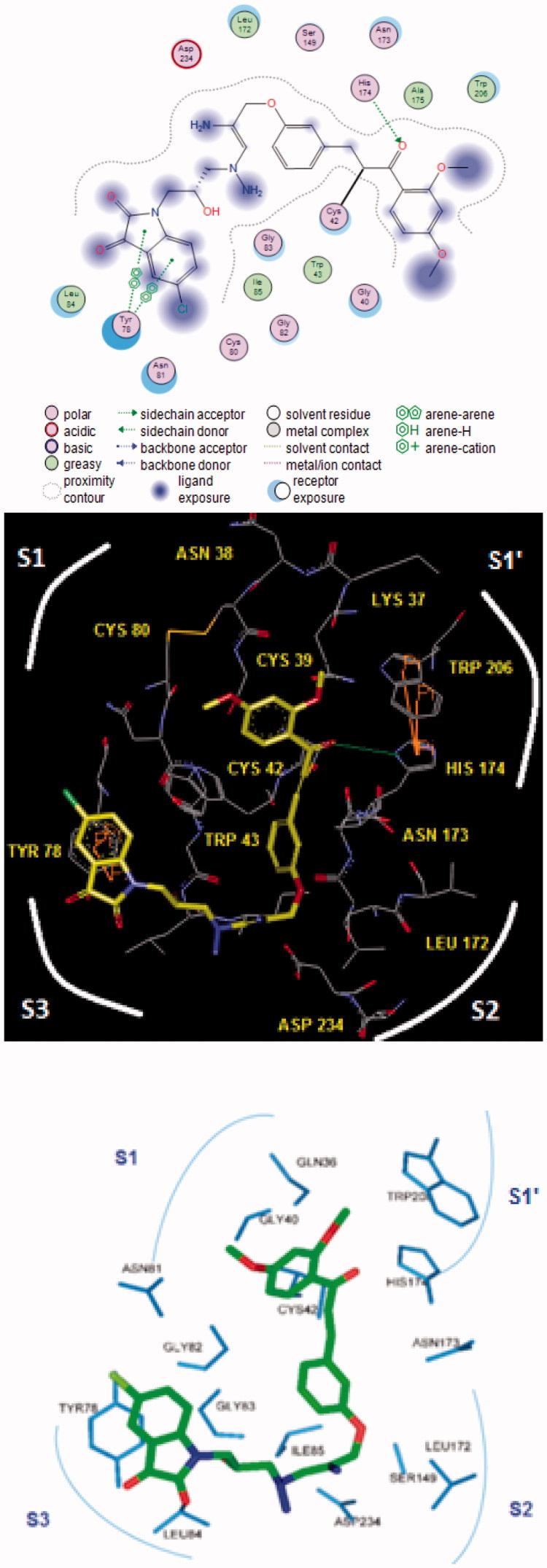
(Top) 2D schematic interaction diagram of the most potent inhibitor HLCIC1 (Table 1) at the active-site of FP-2 of *Pf*; (Middle, Bottom) 3D structure of the active-site with bound HLCIC1.

**Figure 5.**
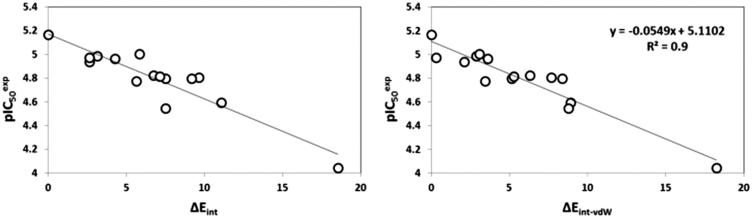
Plot of correlation between p*IC*_50_^exp^ and overall intermolecular interaction energy *E*_int_ in complexes FP-2:HLCIC_x_.

The bound conformation of the most potent FP-2 inhibitor HLCIC1^12^ in this QSAR model is displayed in Figure 4 (Table 1). The enzyme–inhibitor overall intermolecular interaction energy E_int_ in FP-2:HLClCx complexes is listed in Table 4 along with its correlation between that energy with observed inhibitory potency (*IC*_50_^exp^) plotted in Figure 5. According to the statistical data in Table 5, some 85% of the *IC*_50_^exp^ variation is explained by E_int_ drop while moving from the best active HLClC1 to the less active one HLClC6. The quality of this correlation opens the gate to a deeper analysis of the intermolecular interaction energy E_int_ variation in light with structural requirement of FP-2 inhibition namely the active site pockets filling.

**Table 4:** Enzyme–inhibitor FP-2:HLCICx overall intermolecular interaction energy *E*_int_ (kcal·mol^−1^).

Training set^a^	E_vdw_^b^	E_ele_^c^	E_int_^d^	ΔE_int_	pIC_50_^exp^
HLCIC1	−57.32	−2.07	−59.39	0	5.167
HLCIC2	−54.43	−1.83	−56.26	3.13	4.987
HLCIC3	−55.21	−1.53	−56.75	2.64	4.939
HLCIC4	−48.41	0.09	−48.31	11.08	4.594
HLCIC5	−49.63	−0.11	−49.75	9.64	4.807
HLCIC6	−39.08	−1.76	−40.85	18.54	4.043
HLCIC7	−53.71	−1.40	−55.11	4.28	4.964
HLCIC8	−48.53	−3.35	−51.88	7.51	4.545
HLCIC9	−52.19	0.32	−51.87	7.52	4.798
HLCIC10	−48.93	−1.27	−50.21	9.18	4.796
HLCIC11	−51.00	−1.62	−52.63	6.76	4.822
HLCIC12	−52.05	−0.21	−52.26	7.13	4.814
HLCIC13	−54.24	0.68	−53.55	5.84	5.003
HLCIC14	−57.01	0.28	−56.73	2.66	4.974
HLCIC15	−53.87	0.10	−53.76	5.63	4.776

^a^For the chemical structures of the training set of inhibitors see Table 1.

^b^van der Walls component of non-bonded of interaction energy.

^c^Electrostatic component of non-bonded interaction energy.

^d^ *E*_int_ is the interaction energy of two sets of atoms, one set represents residues of the FP-2 the other the inhibitor: *E*_int_ = *E*_vdW_ + *E*_ele_.

^e^ *IC*_50_^exp^ is the experimental half-maximal inhibitory concentration of the HLCIC obtained from reference^12^, p*IC*_50_^exp^ = −log_10_(*IC*_50_^exp^).

**Table 5.** Regression analysis of computed interaction energies *E*_int_ and observed activities p*IC*_50_^exp^ of hybrid lactone–chalcone and isatin–chalcone HLCIC.

Statistical data of linear regression	(C)
$pIC_{50}{}^{\;\text{exp}\;}=-0.0548\cdot \Delta E_{\text{int}}+5.1734$ (C)	
Number of compounds, n	15
Squared correlation coefficient of regression, *R*^2^	0.85
LOO cross-validated correlation coefficient, *R*_xv_^2^	0.83
Standard error of regression, σ	0.106
Statistical significance of regression, Fischer *F*-test	72.10
Level of statistical significance, α	>95%
Range of activities *IC*_50_^exp^ (μM)	6.8–90.0

**Figure 6.**
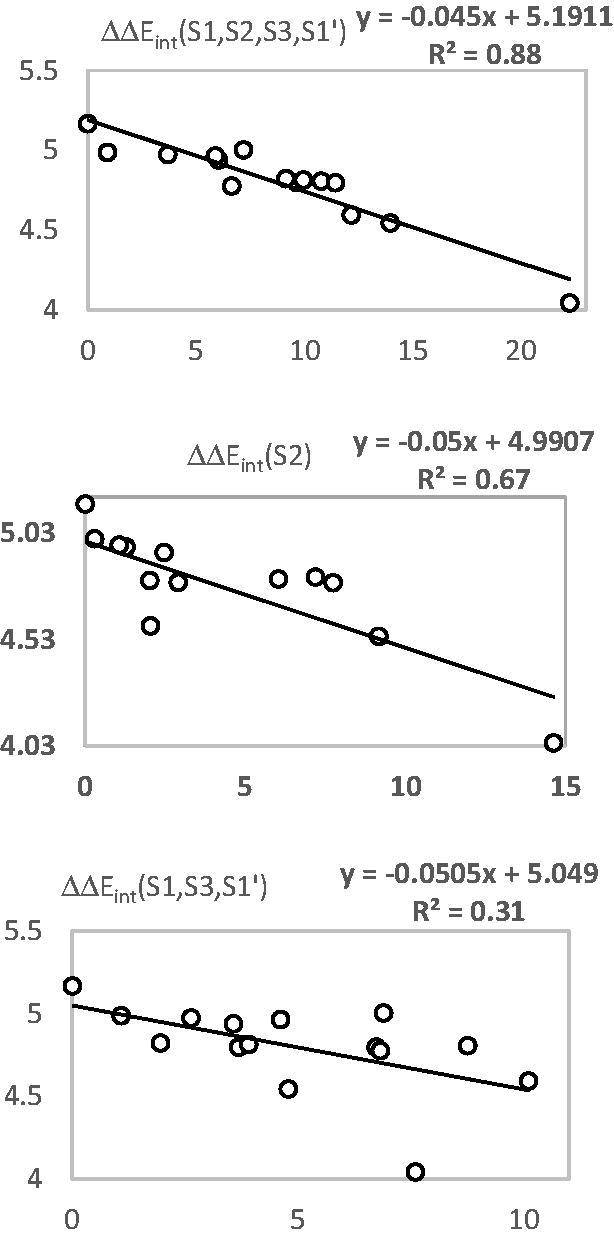
Plot of the correlation between interaction energies of residues belonging to individual active site pockets (S1, S2, S3, and S’1) and observed activities p*IC*_50_^exp^.

The prominent role of the van der Waals (vdW) component of *E*_int_ in the binding affinity of HLCIC to FP-2 of *Pf* is highlighted by the correlation between individual contributions to the overall *E*_int_. In addition, to assess the impact of the residues occupying individual active site pockets (S1, S2, S3, and S1’; Figure 4) we have analyzed their contribution to the overall *E*_int_ (Table 6; Figure 6). The contribution of all the four pockets together explained 88% of the FP-2 inhibitory potencies of the training set inhibitors. This fell down to 31% when removing the contribution of the S2 pocket (67%). The filling of the S2 pocket by function groups of the inhibitors is therefore crucial for the FP-2:HLCIC1-15 affinity. Thus, our virtual FP-2 inhibitor design prioritised optimal filling of the S2 pocket by the HLCIC analogues.

**Table 6.** Active site residue contribution to *E*_int_ in FP-2:HLCIC1 complex (kcal·mol^−1^).

Pockets	pIC_50_^exp^	S1							S2						
Residue		GLN 36	GLY 40	SER 41	CYS 42	CYS 80	ASN 81	Total	LEU 84	ILE 85	SER 149	LEU 172	ALA 175	ASP-234	Total
HLCIC 1	5.17	−1.5	−3.1	−0.7	0.5	−1.2	−3.8	−9.9	−3.7	−1.6	−1.9	−4.0	−2.3	−2.5	−16.0
HLCIC 2	4.99	−0.9	−2.9	−2.9	0.5	−1.0	−3.8	−11.1	−3.7	−1.6	−1.9	−4.0	−2.3	−2.6	−16.1
HLCIC 3	4.94	−1.9	−3.2	−0.9	−0.7	−1.1	−2.5	−10.4	−3.7	−2.1	−1.6	−3.5	−2.4	−0.3	−13.5
HLCIC 4	4.59	−0.4	−0.8	−0.4	1.1	−0.3	−3.4	−4.1	−4.1	−1.4	−1.5	−2.7	−2.2	−2.0	−13.9
HLCIC 5	4.81	−0.4	−0.9	−0.5	1.1	−0.3	−3.5	−4.5	−4.1	−1.4	−1.5	−2.8	−2.2	−1.9	−13.9
HLCIC 6	4.04	−3.1	−3.2	−2.0	−2.6	−1.6	−5.6	−18.2	−0.2	−0.1	−0.1	−0.2	−0.7	−0.1	−1.3
HLCIC 7	4.96	−1.4	−2.5	−0.7	0.4	−1.0	−3.9	−9.1	−3.7	−2.0	−1.8	−3.7	−2.2	−1.2	−14.7
HLCIC 8	4.55	−0.8	−2.0	−0.7	−0.8	−0.9	−4.4	−9.6	−2.7	−1.6	−0.6	−0.3	−1.7	0.0	−6.8
HLCIC 9	4.80	−1.3	−2.7	−1.1	0.8	−1.2	−4.0	−9.5	−3.7	−1.5	−1.6	−3.5	−1.7	−1.0	−13.1
HLCIC 10	4.80	−0.5	−1.3	−1.1	0.4	−0.7	−5.2	−8.4	−2.5	−0.6	−1.0	−2.2	−1.4	−0.5	−8.2
HLCIC 11	4.82	−0.3	−1.2	−0.9	−0.7	−0.7	−5.2	−9.0	−2.6	−0.7	−0.9	−2.4	−1.5	−0.6	−8.8
HLCIC 12	4.81	−2.1	−3.9	−1.3	−0.7	−1.2	−3.0	−12.2	−3.3	−1.0	−0.9	−3.2	−1.5	−0.1	−9.9
HLCIC 13	5.00	−0.7	−1.8	−0.8	0.1	−1.0	−5.8	−10.1	−3.7	−1.7	−2.1	−3.7	−1.7	−2.7	−15.7
HLCIC 14	4.97	−1.2	−3.1	−1.0	0.1	−1.1	−5.1	−11.4	−3.7	−1.6	−2.0	−3.6	−1.8	−2.2	−14.9
HLCIC 15	4.78	−0.9	−2.0	−0.6	1.6	−0.9	−3.9	−6.6	−3.8	−1.6	−2.2	−3.9	−1.9	−2.7	−16.1
Pockets		S3						S1’							
Residue	pIC_50_^exp^	LYS + 76	ASN 77	TYR 78	GLY 82	GLY 83	Total	VAL150	VAL 152	ALA 157	ASN 173	HISD174	TRP 206	ASN 204	Total
HLCIC 1	5.17	−0.4	−0.5	−8.3	−3.0	−3.8	−15.9	−0.5	0.0	0.0	−3.0	−4.6	−2.4	−0.1	−10.7
HLCIC 2	4.99	−0.4	−0.5	−8.3	−2.9	−3.8	−15.8	−0.5	−0.2	0.0	−3.0	−4.2	−1.5	−0.1	−9.5
HLCIC 3	4.94	−0.3	−0.3	−6.2	−2.3	−3.4	−12.6	−0.5	0.0	0.0	−3.6	−4.9	−1.7	−0.1	−10.8
HLCIC 4	4.59	−0.3	−0.4	−7.1	−3.3	−4.0	−15.2	−0.5	0.0	0.0	−4.1	−4.3	−0.2	0.0	−9.2
HLCIC 5	4.81	−0.3	−0.4	−7.2	−3.3	−4.0	−15.2	−0.5	0.0	0.0	−4.4	−4.8	−0.3	0.0	−10.1
HLCIC 6	4.04	0.1	−0.1	−0.3	−2.0	−0.7	−3.0	−0.1	0.0	0.0	−4.2	−1.1	−2.4	0.0	−7.8
HLCIC 7	4.96	−0.4	−0.5	−8.5	−2.8	−4.1	−16.3	−0.5	0.0	0.0	−2.7	−4.5	−0.6	0.0	−8.3
HLCIC 8	4.55	−0.8	−3.2	−2.9	−4.6	−4.2	−15.5	−0.2	0.0	0.0	−2.1	−5.2	−0.7	0.0	−8.3
HLCIC 9	4.80	−0.4	−0.4	−8.2	−3.0	−3.8	−15.9	−0.4	0.0	0.0	−2.7	−3.0	−0.4	0.0	−6.4
HLCIC 10	4.80	−0.9	−0.8	−8.3	−5.7	−5.3	−21.0	−0.5	0.0	0.0	−3.4	−1.7	−0.1	0.0	−5.7
HLCIC 11	4.82	−0.9	−0.8	−8.3	−6.1	−5.4	−21.5	−0.5	0.0	0.0	−3.8	−1.9	−0.1	0.0	−6.3
HLCIC 12	4.81	−0.2	−0.4	−5.5	−3.9	−4.8	−14.8	−0.3	0.0	0.0	−2.9	−3.4	−0.6	0.0	−7.3
HLCIC 13	5.00	−0.4	−0.5	−8.3	−3.0	−3.9	−16.1	−0.4	0.0	0.0	−2.6	−2.0	−0.4	0.0	−5.5
HLCIC 14	4.97	−0.4	−0.5	−8.3	−3.2	−4.1	−16.4	−0.4	0.0	0.0	−2.7	−2.5	−1.4	0.0	−7.0
HLCIC 15	4.78	−0.4	−0.5	−8.3	−3.1	−3.9	−16.1	−0.5	−0.1	−0.2	−4.2	−3.2	−0.5	−0.1	−8.9

**Figure 7.**
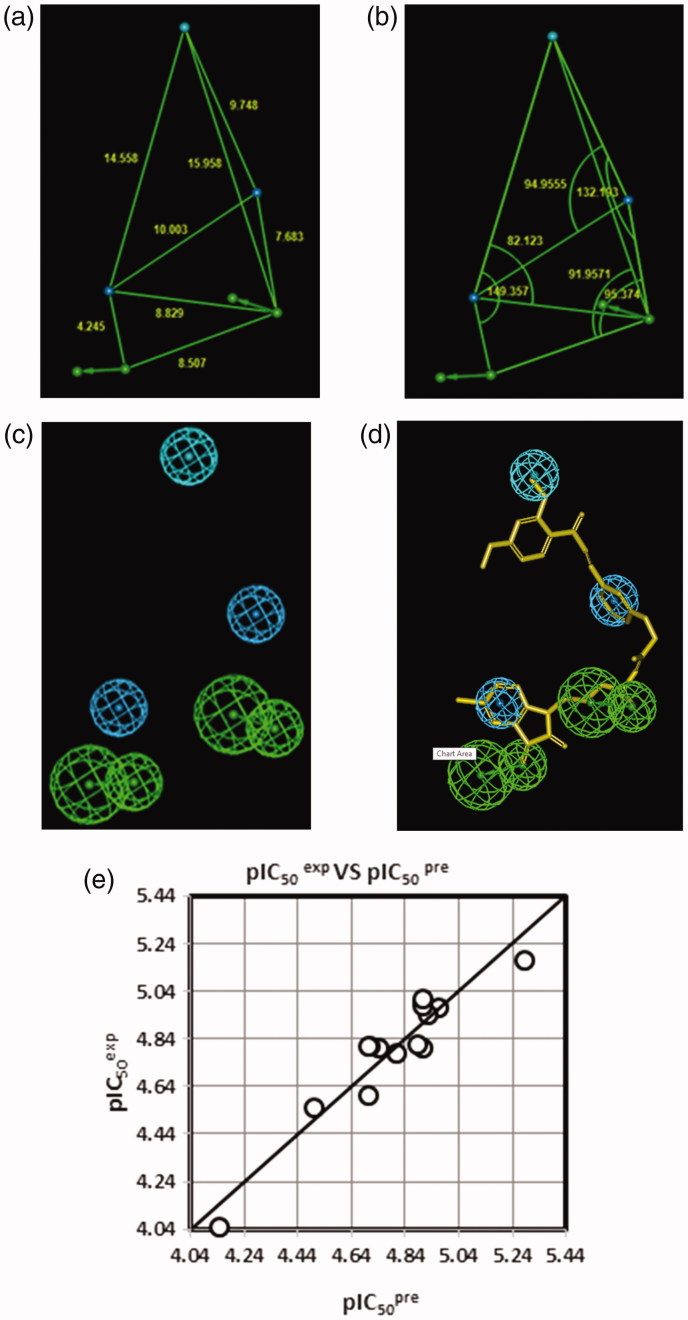
Distances (a), angles (b), features (c), and mapping (d) of the pharmacophore of the *Pf* FP-2 inhibition with the best training set inhibitor HLCIC1 (yellow)^12^. The correlation plot of experimental vs. predicted inhibitory activity (e) is displayed. The features are coloured blue for hydrophobic aliphatic (HYd), green for hydrogen-bond (HB) acceptor (HBA), purple for HB donor (HBD) and orange for Aromatic (Ar). The arrows represent the projection of donor and acceptor features.

The PH4 pharmacophore model of FP-2 inhibition elaborated from QSAR model and training set of HLCIC^12^ is presented in Tables 7 and 8 and Figure 7. The 3D-QSAR PH4 generation was carried out in three steps: constructive, subtractive, and optimisation step (Section 2). During the constructive phase of HypoGen the most active HLCIC, for which *IC*_50_^exp^ ≤ 2 × 6.8 µM, was selected as the leads. Thus, HLCIC1-3,7,13,14 (*IC*_50_^exp^ ≤ 13.6 µM) were used to generate the starting PH4 features and those matching these leads were retained. During the subsequent subtractive phase, features which were present in more than half of the inactive HLCIC were removed. The PH4 models which contained all features were retained. None of the training set compounds was found to be inactive (*IC*_50_^exp^> 6.8 × 10^3.5^ = 21503.4 µM). During the final optimisation phase, the score of the PH4 hypotheses was improved. Hypotheses were scored via simulated annealing protocol according to errors in the activity estimates from the regression and complexity. At the end of optimisation, 10 best scoring unique hypotheses (Table 7) displaying five features were kept. The reliability of the generated PH4 models was then assessed using the calculated cost parameters ranging from 74.8 (Hypo1) to 94.7 (Hypo10). Their statistical data (costs, root-mean-square deviation 2.056 ≤ *RMSD* ≤ 2.777 and 0.89 ≤ *R* ^2^ ≤ 0.94) are listed in Table 7. The PH4 Hypo1, with the best *RMSD* and highest *R* ^2^ was retained for screening. Its regression equation p*IC*_50_^exp^ = 0.9958·p*IC*_50_^pre^ + 0.0192 (Figure 7; Table 8), both *R* ^2^ and *R*_xv_^2^ greater than 0.9 and *F*-test of 111.25 attest the predictive capacity of the PH4. The fixed cost of Hypo1 (74.8), lower than the null cost (296.7) by Δ = 221.9, is a chief indicator of the PH4 model predictability (Δ > 70 corresponds to a probability higher than 90% that the model represents a valid correlation^22^). The difference Δ ≥ 202 for the set of 10 hypotheses confirms the high quality of the PH4 model. The best-selected hypothesis Hypo1 represents a PH4 model with a similar level of predictive power as the QSAR model utilising the GFE of E:I complex formation with a probability of 98%.

**Table 7.** Output parameters of 10 generated PH4 hypotheses for test set HLCIC FP-2 inhibitors^12^ after CatScramble validation procedure.

Hypothesis	*RMSD*^a^	*R*^2^^b^	Total cost^c^
Hypo1	2.056	0.94	74.8
Hypo2	2.368	0.92	80.7
Hypo3	2.463	0.91	83.5
Hypo4	2.3	0.92	84.5
Hypo5	2.574	0.90	86.3
Hypo6	2.614	0.90	88.4
Hypo7	2.624	0.90	89.6
Hypo8	2.738	0.89	91.8
Hypo9	2.682	0.89	92.1
Hypo10	2.777	0.89	94.7
Fixed cost	0	1	30.9
Null cost	6.142	0	296.7

^a^Root mean square deviation.

^b^Squared correlation coefficient.

^c^Overall cost parameter of the PH4.

**Table 8.** Regression analysis of p*IC*_50_^exp^ ^12^ and computed p*IC*_50_^pre^ of HLCIC towards the FP-2 of *Pf*.

Statistical data of linear regression for Hypo 1 (D)		
$pIC_{50}{}^{\text{exp}\;}=0.9958\cdot pIC_{50}{}^{\text{pre}}+0.0192$ (D)		
Number of compounds, n	15	
Squared correlation coefficient of regression, *R*^2^	0.89	
LOO cross-validation squared correlation coefficient, *R*_xv_^2^	0.88	
Standard error of regression, *σ*	0.088	
Statistical significance of regression, Fischer *F*-test	111.249	
Level of statistical significance, *α*	>95%	
Range of activities *IC*_50_^exp^ (μM)	[6.8–90]	

The substantial predictive power of the generated PH4 model was also checked through the computed ratio of PH4-predicted and experimentally observed activities (p*IC*_50_^pre^/p*IC*_50_^exp^) for the VS, (Table 1). The computed ratios are as follows: HLCIV1:1.006, HLCIV2:1.057, HLCIV3:0.97; all of them close to one.

We have built a VL of new HLCIC analogues compounds with a variety of substitutions in ortho, meta, and para positions of the benzene rings with the goal to identify more potent orally bioavailable inhibitors of the FP-2 of *Pf*.

During the VL enumeration, the *R*-groups listed in Table 9 were attached to positions *R*_1_–*R*_6_ of the HLCIC scaffold to form a virtual combinatorial library of the size: R1 × R2 × R3 × R4 × R5 × R6 = 128 × 10 × 128 × 2 × 2 × 2 = 1,310,720 analogues for the scaffold SC1 (Table 9) and R1 × R2 × R3 × R4 × R5 × R6 = 2 × 2 × 2 × 128 × 10 × 28 = 1,310,720 for SC2 (Table 9) resulting together in 1,310,720 × 2 = 2,621,440 analogues. In order to match the substitution pattern of the best training set inhibitor HLCIC1 and taking into account the reported structural information about S pockets filling suitable for substitution^12^ not excluded through the Lipinski’s rule violation (*M_w_* > 500 g/mol)^41^, the VL underwent a focusing.

**Table 9.** *R*-groups (fragments, building blocks, substituents) used in the design of the diversity VL of HLCIC analogues.

*R*_1_, *R*_2_, *R*_3_, *R*_4_, *R*_5_ and *R*_6_-groups ^a,b^		linker-isatin (-lki)	
R-group			
1. –F	2. –Cl	3. –Br	4. –I
5. –OH	6. –SH	7. –NH_2_	8. –OCH_3_
9. –OCl	10. –OBr	11. –OI	12. –OSH
13. –OCH_2_OH	14. –OCH_2_Cl	15. –OCH_2_Br	16. –OCH_2_F
17. –OCH_2_I	18. –OCH_2_SH	19. –OCH_2_NH_2_	20. –OCH_2_COO–
21. –OCH_2_COOH	22. –OCH_2_CHO	23. –OCH_2_CN	24. –OCH_2_C(NH_2_)_2_^+^
25. –OCH_2_CONH_2_	26. –OCH_2_NO_2_	27. –OCH_2_SO_2_	28. –OCH_2_PO_3_H
29. –(CH_2_)_3_NH_2_	30. –(CH_2_)_3_OH	31. –(CH_2_)_3_Cl	32. –(CH_2_)_3_Br
33. –(CH_2_)_3_F	34. –(CH_2_)_3_I	35. –(CH_2_)_3_COO–	36. –(CH_2_)_3_COOH
37. –(CH_2_)_3_CHO	38. –(CH_2_)_3_CN	39. –(CH_2_)_3_C(NH_2_)_2_^+^	40. –(CH_2_)_3_CONH_2_
41. –(CH_2_)_3_NH_2_CO	42. –(CH_2_)_3_NO_2_	43. –(CH_2_)_3_SO_2_H	44. –(CH_2_)_3_PO_3_H
45. cycloprop-2-enyl	46. 2-hydroxycycloprop-2-en-1-yl	47. 2,3-dihydroxycycloprop-2-enyl	48. 2-amino-3-hydroxycycloprop-2-enyl
49. 2,3-diaminocycloprop-2-enyl	50. 2-amino-3-fluorocycloprop-2-enyl	51. 2,3-difluorocycloprop-2-enyl	52. 2-chloro-3-fluorocycloprop-2-enyl
53. 2,3-dichlorocycloprop-2-enyl	54. 2-chloro-3-mercaptocycloprop-2-enyl	55. 2*H*-azirin-2-yl	56. 3*H*-diazirin-3-yl
57. 2*H*-triazirin-2-yl	58. 1*H*-tetrazol-5-yl	59. 1,4-dioxan-2-yl	60. –COO–
61. –COOH	62. –CHO	63. –CN	64. –C(NH_2_)+
65. –CONH_2_^+^	66. –NCH_2_O	67. –NO_2_	68. –SO_2_
69. –PO_3_H	70. –C_2_SO_2_H	80. –HSO_2_	81. –CF_3_
82. –CCl_3_	83. –CH_2_CF_3_	84. –CH_2_CCl_3_	85. –(CH_2_)_2_CCl_3_
86. –(CH_2_)_2_CF_3_	87. –(CH_2_)_2_F	88. –N = NH	89. –ON = NH
90. –ON = NOH	91. –N = NOH	92. –NO	93. –CH = CH_2_
94. –CH_2_–CH = CH_2_	95. –(CH_2_)4F	96. –CH_2_–CH = C(NH_2_)_2_	97. –CH_2_–CH = C(OH)NH_2_
98. –CH_2_–CH = C(OH)_2_	99. –CH_2_–CH = CHOH	100. –(CH_2_)_4_OH	101. –(CH_2_)_4_NH_2_
102. –CH_2_–CH = CH–CH_2_OH	103. –CH_2_–CH = CH–CH_2_NH_2_	104. –CH_2_–CH = CH–CH_2_NCO	105. –CH_2_–CH = CH–CH_2_NO_2_
106. –H_2_–CH = CH–CH_2_F	107. –CH_2_–CH = CH–CH_2_COO–	108. –CH_2_–CH = CH–CH_2_COOH	109. –CH_2_–CH = CH–CH_2_CO
110. –CH_2_–CH = CH–CH_2_CN	111. –CH_2_–CH = CH–CH_2_C(NH_2_)_2_^+^	112. –CH_2_–CH = CH–CH_2_CONH_2_	113. –CH_2_–CH = CH–CH_2_SO_2_
114. –CH_2_–CH = CH–CH_2_–PO_3_	115. –CH = C(OH)_2_	116. –CH_2_–CH = C(NH_2_)NO_2_^2−^	117. –CH_2_–CH = C(NO_2_^2−^)_2_
118. –(CH_2_)_4_COOH	119. –CH_2_–CH = CH–CH_2_–NH–CHO	120. –CH_2_–CH = CH–(CH_2_)_2_–CN	121. 3-(2,3-difluorocycloprop-2-enyl) propyl
122. 3,4-difluorofuran-2-yl	123. –(2-CN-6-MeO-1,2,5-triazin-4-yl)Me	124. –(2-cyano-pyrimidin-4-yl)Me	125. 2-cyano-pyrimidin-4-yl
126. (pyrimidin-4-yl)Me	127. pyrimidin-4-yl	128. (tetrahydro-2*H*-pyran)Me	129. (piperidin-4-yl)Me
130. (isopropyl-piperidin-4-yl)Me	131. (tetrahydrofuran-2-yl) ethyl	132. –CH(Me)_2_	133. –CH_2_–CH(Me)–CH_3_
134. –CH_2_–CH(Me)–(CH_2_)_2_OH	135. –CH_2_–CH(Me)–(CH_2_)_2_NH_2_	136. –CH_2_–CH(Me)–(CH_2_)_2_Cl	137. –CH(CH_2_)_2_–CH_3_ 138. –lki

^a^Fragments 1–128 were used in *R*_1_-groups and *R*_3_-groups; fragments 1–10 were used in *R*_2_-group, fragments 138 and H for *R*_4_, R_5_, and *R*_6_ as scaffold SC1. Reversely fragments 1–128 were used in *R*_6_-groups and *R*_4_-groups; fragments 1–10 were used in *R*_5_-group, fragments 138 and H for *R*_3_, R_2_, and *R*_1_ as scaffold SC2. Fragments 129–138 were used intuitively as *R*_6_-groups substituents for the best VL hits at P2 position according to Schechter and Berger notation^42^.

^b^(–) bond indicates the attachment points of individual fragments.

To increase the content of drug-like and orally bioavailable analogues, the initial VL was filtered in an ADME-based focusing step. Only those molecules that satisfied the Lipinski’s rule of five^41^ computed using QikProp^16^, were kept.

From the initial set of 2,621,440 (1,310,720 × 2) analogues, 18,288 (9144 × 2) fulfilled the Lipinski test (except the restriction *M_w_* < 500 g/mol). Out of them, 141 analogues mapped to the 5 feature PH4 pharmacophore. The 81 best fitting analogues (PH4 hits) were retained and submitted to structure-based screening using the QSAR model and computed GFE of the FP-2:HLCIC complex formation. The calculated ΔΔ*G*_com_ of the FP-2:HLCIC complexes of the hits, their components as well as predicted half-maximal inhibitory concentrations *IC*_50_^pre^ estimated from the correlation equation (B) (Table 3) are listed in Table 10. Thirty-three others HLClC new analogues were added from an intuitive substitution approach intended to fill better the enzyme S2 pocket; they are listed in Table 11. In the majority of new HLCIC analogues, the estimated inhibitory potencies are better than that for the most active training set inhibitor HLCIC1 (*IC*_50_^exp^ = 6.8 µM)^12^.

**Table 10.** GFE of FP-2:HLCIC complex formation and its components for the 81 virtually designed HLCIC exploring the pockets S1, S1’, and S3.


No	Analogues	ΔΔ*H*_MM_^a^	ΔΔ*G*_sol_^b^	ΔΔ*TS*_vib_^c^	ΔΔ*G*_com_^d^	*IC*_50_^pre^^e^
	HLCIC1	0	0	0	0	6800^f^
	Ortho 1 (o.SC1)					

1	33–5–4–lki–H–H	15.57	−1.88	1.08	12.59	16410
2	31–2–30–lki–H–H	10.97	−1.13	−0.53	10.38	14550
3	31–H–100–lki–H–H	10.46	−2.53	4.47	3.44	9980
4	30–6–1–lki–H–H	12.32	−1.22	0.34	10.76	14850
5	34–6–102–lki–H–H	9.70	−1.11	3.78	4.80	10740

No	Meta 1 (m.SC1)	ΔΔ*H*_MM_	ΔΔ*G*_sol_	ΔΔ*TS*_vib_	ΔΔ*G*_com_	*IC*_50_^pre^

6	51–1–1–H–lki–H	−67.28	1.49	−11.50	−54.29	430
7	2–8–83–H–lki–H	−47.00	0.08	−4.35	−42.55	810
8	45–1–99–H–lki–H	−44.27	0.76	−0.34	−43.14	790
9	H–1–55–H–lki–H	−60.80	3.67	−10.46	−46.66	650
10	H–H–5–H–lki–H	−44.12	−0.47	−4.68	−39.90	940
11	4–5–1–H–lki–H	−45.96	−0.27	−6.09	−40.14	930
12	33–H–102–H–lki–H	−49.75	0.57	1.00	−50.18	540
13	33–1–99–H–lki–H	−49.55	0.76	−3.08	−45.69	690
14	14–6–1–H–lki–H	−48.66	−0.27	−6.42	−42.51	820
15	14–H–2–H–lki–H	−46.76	0.12	−5.41	−41.22	880
16	33–1–37–H–lki–H	−47.22	2.17	−0.48	−44.56	730
17	33–H–17–H–lki–H	−46.92	0.63	−0.52	−45.76	680
18	12–H–52–H–lki–H	−39.45	1.21	−6.61	−31.61	1480
19	31–1–63–H–lki–H	−53.54	−0.31	−3.02	−50.84	520
20	31–7–4–H–lki–H	−49.53	−0.04	−2.20	−47.38	630
21	29–2–106–H–lki–H	−45.44	1.56	1.92	−45.80	680
22	12–1–106–H–lki–H	−47.88	0.37	−3.25	−44.25	740
23	18–H–106–H–lki–H	−46.70	0.65	−1.26	−44.78	720
24	16–1–106–H–lki–H	−49.96	0.07	−3.50	−46.38	660
25	53–2–2–H–lki–H	−76.38	1.37	−13.87	−61.12	290
26	122–1–1–H–lki–H	−95.43	1.53	−10.6	−83.3	90
27	121–1–1–H–lki–H	−68.89	0.16	−9.19	−59.53	320
28	53–H–2–H–lki–H	−75.66	0.92	−11.97	−62.76	270
29	53–2–3–H–lki–H	−77.01	1.15	−13.81	−62.04	280
30	124–1–1–H–lki–H	−66.93	−4.80	−2.48	−69.24	190
31	125–1–1–H–lki–H	−100.28	−4.18	−1.85	−102.61	30
32	126–1–1–H–lki–H	−71.57	−2.25	0.20	−74.03	150
33	127–1–1–H–lki–H	−108.48	−0.47	−2.86	−106.09	30

No	Para 1 (p.SC1)	ΔΔ*H*_MM_	ΔΔ*G*_sol_	ΔΔ*TS*_vib_	ΔΔ*G*_com_	*IC*_50_^pre^

34	32–1–101–H–H–lki	−0.53	0.31	5.30	−5.51	6130
35	31–2–62–H–H–lki	2.24	−0.80	0.12	1.31	8890
36	52–H–101–H–H–lki	−0.57	0.05	−0.43	−0.08	8230
37	53–1–29–H–H–lki	−4.13	1.19	−0.29	−2.64	7170
38	4–8–31–H–H–lki	1.75	−0.12	−0.65	2.29	9370
	Ortho 2 (o.SC1)					

39	lki–H–H–33–4–29	−41.31	−3.01	1.85	−46.18	670
40	lki–H–H–16–1–32	−42.10	−3.74	−2.16	−43.68	770
41	lki–H–H–33–H–37	−40.09	−1.69	0.12	−41.90	840
42	lki–H–H–15–H–1	−38.16	−3.96	−5.84	−36.28	1 150
43	lki–H–H–9–1–93	−36.42	−5.15	−3.76	−37.81	1 060
44	lki–H–H–29–H–2	−36.90	−2.59	0.49	−39.98	940
45	lki–H–H–18–H–31	−41.11	−4.09	0.19	−45.40	700
46	lki–H–H–33–H–38	−41.06	−2.68	−0.62	−43.12	790
47	lki–H–H–53–H–30	−43.70	−4.14	−2.52	−45.33	700
48	lki–H–H–4–6–93	−38.33	−2.03	−4.01	−36.34	1 140
49	lki–H–H–2–5–31	−38.76	−4.17	−1.63	−41.30	870
50	lki–H–H–4–7–32	−38.83	−5.59	−4.58	−39.84	940
51	lki–H–H–4–6–38	−38.95	−3.98	−1.80	−41.13	880
52	lki–H–H–1–6–H	−35.94	−4.94	−5.63	−35.25	1210
53	lki–H–H–1–2–102	−39.53	−2.57	−0.92	−41.18	880
54	lki–H–H–6–7–31	−37.56	−4.91	−1.72	−40.75	900
55	lki–H–H–2–H–29	−37.12	−4.87	−2.10	−39.89	940
56	lki–H–H–1–4–106	−53.16	−3.05	−6.37	−49.84	550

No	Meta 2 (m.SC2)	ΔΔ*H*_MM_	ΔΔ*G*_sol_	ΔΔ*TS*_vib_	ΔΔ*G*_com_	*IC*_50_^pre^
57	H–lki–H–33–4–29	−45.50	−1.61	−0.12	−46.99	640
58	H–lki–H–33–H–37	−46.73	−0.43	0.98	−48.15	600
59	H–lki–H–15–H–1	−44.73	−1.07	−6.52	−39.28	970
60	H–lki–H–29–H–2	−44.41	−1.59	0.32	−46.33	660
61	H–lki–H–18–H–31	−43.73	−0.95	−3.32	−41.36	870
62	H–lki–H–33–H–38	−45.55	−1.57	−0.28	−46.85	640
63	H–lki–H–16–6–95	−47.00	−1.36	−1.35	−47.01	640
64	H–lki–H–50–H–31	−45.63	0.42	−3.10	−42.10	830
65	H–lki–H–H–1–46	−42.57	−1.87	−4.77	−39.67	950
66	H–lki–H–H–7–31	−40.87	−2.81	−1.01	−42.66	810
67	H–lki–H–51–H–30	−48.12	−2.20	0.68	−51.00	510
68	H–lki–H–1–1–29	−39.50	−2.10	−1.34	−40.27	920
69	H–lki–H–4–6–93	−42.95	−1.69	−3.28	−41.37	870
70	H–lki–H–2–5–31	−41.22	−1.24	−2.76	−39.69	950
71	H–lki–H–4–7–32	−42.49	−1.60	−4.56	−39.52	960
72	H–lki–H–4–6–38	−42.63	−2.52	−4.73	−40.41	920
73	H–lki–H–1–2–102	−42.14	−0.83	−3.42	−39.55	960
74	H–lki–H–6–7–31	−41.44	−1.53	−2.26	−40.72	900
75	H–lki–H–2–H–29	−42.23	−1.72	−3.29	−40.65	900
76	H–lki–H–1–4–106	−44.34	−1.22	−5.99	−39.57	960
No	Para 2 (p.SC2)	ΔΔ*H*_MM_	ΔΔ*G*_sol_	ΔΔ*TS*_vib_	ΔΔ*G*_com_	*IC*_50_^pre^
77	H–H–lki–45–8–106	3.62	−0.94	−1.54	4.22	10410
78	H–H–lki–52–H–1	0.38	−2.85	−4.56	2.09	9270
79	H–H–lki–52–6–62	1.93	−1.28	−3.36	4.00	10290
80	H–H–lki–4–8–4	0.29	−1.97	−6.02	4.33	10480
81	H–H–lki–1–5–94	4.80	−1.67	1.31	1.81	9130

^a^ΔΔ*H*_MM_ (kcal⋅mol^−1^) is the relative enthalpic contribution to the GFE change of FP-2:HLCIC complex formation ΔΔ*G*_com_ (for details see footnote of Table 2).

^b^ΔΔ*G*_sol_ (kcal⋅mol^−1^) is the relative solvation GFE contribution to ΔΔ*G*_com_.

^c^ΔΔ*TS*_vib_ (kcal⋅mol^−1^) is the relative entropic (vibrational) contribution to ΔΔ*G*_com_.

^d^ΔΔ*G*_com_ (kcal⋅mol^−1^) the relative GFE change of the FP-2:HLCIC complex formation ΔΔ*G*_com_ = ΔΔ*H*_MM_ + ΔΔ*G*_sol_ + ΔΔ*TS*_vib_.

^e^ *IC*_50_^pre^ (nM) is the predicted half-maximal inhibitory concentration of HLCIC towards FP-2 of *Pf* calculated from ΔΔ*G*_com_ using correlation equation (B) (Table 3).

^f^ *IC*_50_^exp^ is given for the reference inhibitor HLCIC1^12^ instead of *IC*_50_^pre^ (nM).

**Table 11.** GFE of FP-2:HLCIC complex formation and its components for the 33 virtually designed HLCIC with intuitive P2 substitution (*R*_6_-group) exploring the S2 pocket in addition to S1, S1’, and S3.

No	Analogues	ΔΔ*H*_MM_	ΔΔ*G*_sol_	ΔΔ*TS*_vib_	ΔΔ*G*_com_	*IC*_50_^pre^
	HLCIC1	0	0	0	0	6800
82	125–1–1–H–lki–128	−113.67	−2.72	2.83	−119.23	13
83	125–1–1–H–lki–129	−111.20	−1.73	3.26	−116.20	15
84	125–1–1–H–lki–134	−110.06	−1.39	1.87	−113.33	18
85	126–1–1–H–lki–128	−84.96	0.07	3.59	−88.48	68
86	126–1–1–H–lki–129	−84.46	0.97	3.18	−86.66	75
87	126–1–1–H–lki–134	−79.04	0.83	3.95	−82.16	95
88	127–1–1–H–lki–128	−119.71	2.17	1.45	−118.99	13
89	127–1–1–H–lki–129	−117.15	2.79	2.60	−116.97	15
90	127–1–1–H–lki–134	−113.51	2.86	3.15	−113.80	17
91	53–2–2–H–lki–128	−76.09	1.53	−4.86	−69.70	187
92	53–2–2–H–lki–129	−73.74	1.93	−2.96	−68.86	196
93	122–1–1–H–lki–130	−107.89	0.72	−1.05	−106.12	26
94	53–H–2–H–lki–128	−82.57	2.57	−1.77	−78.22	118
95	53–H–2–H–lki–136	−72.35	2.61	−4.93	−64.81	245
96	51–1–1–H–lki–128	−76.64	1.84	−2.59	−72.22	164
97	51–1–1–H–lki–133	−68.35	1.15	−2.93	−64.27	252
98	33–H–102–H–lki–131	−74.65	3.54	5.88	−77.00	126
99	33–H–102–H–lki–134	−70.26	3.83	6.98	−73.41	153
100	31–1–63–H–lki–128	−67.37	1.83	0.36	−65.91	230
101	31–1–63–H–lki–129	−65.27	2.31	−0.70	−62.26	281
102	51–H–30–H–lki–131	−80.47	−1.39	2.96	−84.82	82
103	51–H–30–H–lki–136	−81.95	−0.23	4.15	−86.33	76
104	31–H–100–lki–H–128	−73.53	0.58	9.39	−82.34	94
105	31–H–100–lki–H–129	−65.73	0.96	9.24	−74.02	148
106	31–H–100–lki–H–132	−67.83	2.41	1.39	−66.81	219
107	1–4–106–lki–H–131	−70.26	2.91	−0.52	−66.84	219
108	1–4–106–lki–H–133	−61.56	2.57	−0.44	−58.55	344
109	125–1–1–H–lki–132	−108.80	−0.52	−4.86	−104.47	29
110	125–1–1–H–lki–133	−110.12	−2.09	−0.96	−111.26	20
111	125–1–1–H–lki–137	−114.41	−1.70	−0.29	−115.82	16
112	127–1–1–H–lki–132	−113.82	3.33	−1.74	−108.76	23
113	127–1–1–H–lki–133	−115.25	2.25	−1.23	−111.77	19
114	127–1–1–H–lki–137	−120.03	2.59	−1.40	−116.04	15

**Figure 8.**
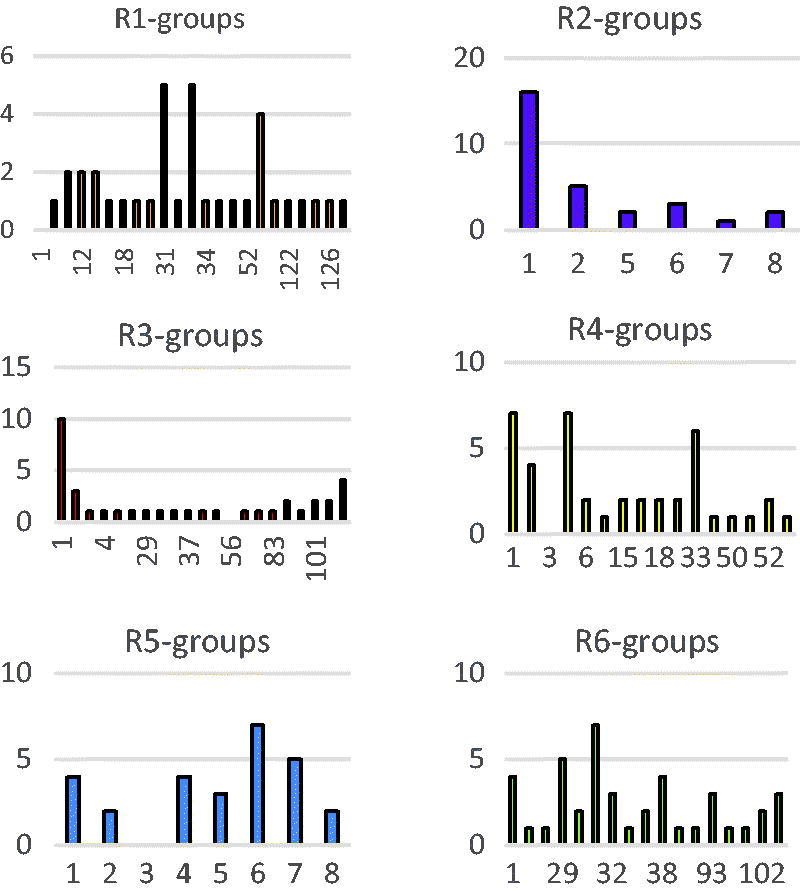
Histograms of frequency of occurrence of individual *R*_1_–*R*_6_ groups in the 81 selected analogues mapping to the five-feature pharmacophore hypothesis Hypo1 (for fragments numbering see Table 9).

In order to identify which substituents from Table 9 lead to new inhibitor candidates with high predicted potencies towards the FP-2, we have prepared histograms of the frequency of occurrence of the *R*_1_ to *R*_6_ groups in these 81 HLCIC virtual hits (Figure 8). Analysis of the histograms showed that the highest frequency of occurrence among the *R*_1_-groups displayed the fragments 31, 33, and 53 (Table 9). In case of *R*_2_-groups fragments 1 and 2; for the *R*_3_-groups fragments 1, 2, and 106; for the *R*_4_-groups fragments 1, 2, 4, and 33 against 1 (4), 4 (4), 6 (7), and 7 (5) for R5 while the R6 group include chiefly fragments 1 (4), 29 (5), 31 (7), and 38 (4).

### 3.2. Discussion

Training set of 15 HLCIC inhibitors and observed half-maximal inhibitory concentrations *IC*_50_^exp^ ^12^ were employed to derive QSAR model of FP-2 inhibition, which uses a single descriptor determined by molecular modelling (GFE of FP-2:HLCIC complex formation, ΔΔ*G*_com_) and crystal structure of the FP-2 of *Pf* in complex with epoxysuccinate E64 (3BPF)^15^. This statistically significant QSAR model confirmed the validity of our 3D models of HLCIC inhibitors and the mode of their binding to the active site of the FP-2 of *Pf*. Then a 3D pharmacophore (PH4) model of FP-2 inhibition was prepared for the bound conformations of HLCIC and was further used for screening of a large virtual combinatorial library of HLCIC analogues with the aim to identify more potent and bioavailable FP-2 inhibitors. The activities of the identified putative inhibitors and analogues proposed by structure-based design (*IC*_50_^pre^) were predicted by linear QSAR regression equation (B) (Table 3) and cross-checked by the PH4 regression equation (D) (Table 8).

#### 3.2.1. QSAR model

The robustness of the single descriptor QSAR model was analyzed by assessing the role of the individual components of GFE of the FP-2:HLCIC complex formation ΔΔ*G*_com_, namely the enthalpic, solvation and approximate entropic contributions. The relevance of the enthalpic contribution ΔΔ*H*_MM_ to GFE was well confirmed by the quality of the regression (A) (Table 3), indicating that a large part (91%) of the variation of the *IC*_50_^exp^ can be explained by intermolecular interactions, which can be traced back to contributions of active site pockets (Sn) as well as individual residues, *E*_int_ (Table 6; Figure 6). Addition of the solvation term ΔΔ*G*_sol_ maintained the relationship level between the experimental data and the modelling results. Finally, the validity of the model was increased by adding the ΔΔ*TS*_vib_ term that describes the loss of the inhibitor vibrational entropy upon enzyme binding, which explained 93% of the variation of the *IC*_50_^exp^. This last contribution is considered to be one of the most reliable indicators of the predictive power of QSAR models as reported by Freire et al.^43^. The VS of 3 HLCICV inhibitors not included into the training set (Table 2) confirmed good correlation between the ΔΔ*G*_com_ and the observed activities *IC*_50_^exp^ ^12^ since the ratio between computed and experimental potencies p*IC*_50_^pre^/p*IC*_50_^exp^ was close to one. Therefore, the QSAR correlation equation (B) and computed relative GFE ΔΔ*G*_com_ can be used for prediction of inhibitory potencies *IC*_50_^pre^ of new HLCIC analogues against the FP-2 of *Pf*, since they share the same binding mode as the training set^12^.

#### 3.2.2. Binding mode of FP-2 inhibitors

Besides the robustness of the QSAR model, the analysis of the interactions between the HLCIC and active site residues revealed the key interactions responsible for the HLCIC affinity to FP-2, such as hydrogen bonds, van der Waals interactions, hydrophobic contacts, etc. As displayed in the 2D and 3D schemes of Figure 4, the binding of HLCIC1 to the active site of FP-2 is supported by the following interactions: HB with His174 and stacking interaction with Tyr78. To verify whether also other stronger interactions co-determine the binding mode of HLCIC to FP-2 active site and aid structure-based design of new analogues, interaction energies *E*_int_ between active site residues and HLCIC were computed (Table 6). The peptidyl structure of HLCIC shed some light on the structural features required for the binding affinity improvement, taking advantage of the S pockets filling (Figure 6). The computed overall interaction energy complexes FP-2:HLCICx correlated with the observed inhibitory potencies *IC*_50_^exp^ of the training set HLCIC inhibitors (Figure 5; Table 5). In particular, the correlation between the van der Waals component of *E*_int_ and *IC*_50_^exp^ pointed to the π–π stacking interaction involving the isatin moiety of the inhibitor and Tyr78.

The S2 pocket filling and interaction energy contribution shows dominant effect on the FP-2 inhibition as reported previously^10^. On the other hand, comparison of the contributions to *E*_int_ between the most active inhibitor HLCIC1 and less active one HLCIC6, corresponds to the trend of activities, but cannot explain the large gap in their inhibitory potencies (1224%). However, in a recent work, we succeeded in justifying the observed 37.5% jump in experimental biological activity between methylphosphonic arginine and hydroxamic acid derivative, both *Pf* Leucyl aminopeptidase (*Pf*A-M17) inhibitors by the enzyme active site residues contribution to *E*_int_ at a level of 35%^44^. Therefore, essential structural information needed for the design of novel potent HLCIC analogues was derived from a more predictive descriptor ΔΔ*G*_com_. New inhibitor candidates were selected according to the workflow (Figure 2), by virtual screening from a diverse VL of analogues with the active site pockets filling as the central structural requirement displayed by the pharmacophore model of FP-2 inhibition provided by the one descriptor (GFE) QSAR model (Table 3; Figure 3).

#### *3.2.3. Analysis of new inhibitors from* in silico *screening*

An analysis of structural requirement for FP-2 inhibition at the level of hydrophobic contacts with the active site revealed that the P2 substituent, namely the *R*_6_-group in the training set insufficiently explored the S2 pocket of the FP-2 active site. Therefore, new HLCIC analogues that match the FP-2 inhibition pharmacophore and fill better the S2 pocket may form potent FP-2 inhibitors (Table 11).

**Figure 9.**
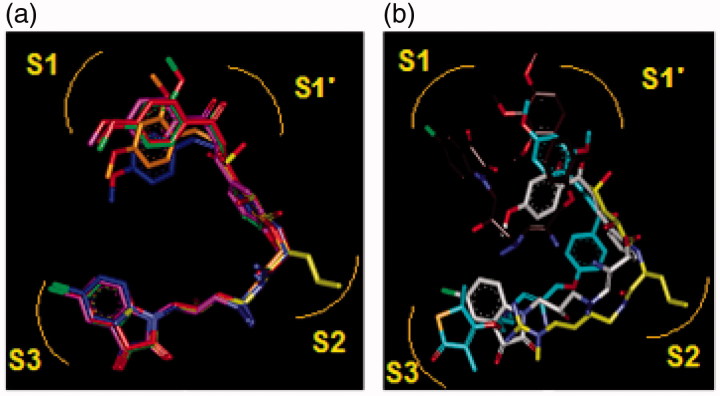
(a) Superposition of most active training set HLClC inhibitors in bound conformation to crystallographic E64 (E64-RX: yellow; HLCIC1: green; HLCIC2: red; HLCIC7: violet; HLCIC13: blue; HLCIC14: orange). (b) Same superposition of less active training set HLClC (E64-RX: yellow; HLCIC4: white; HLCIC8: cyan; HLCIC6: brown).

**Figure 10.**
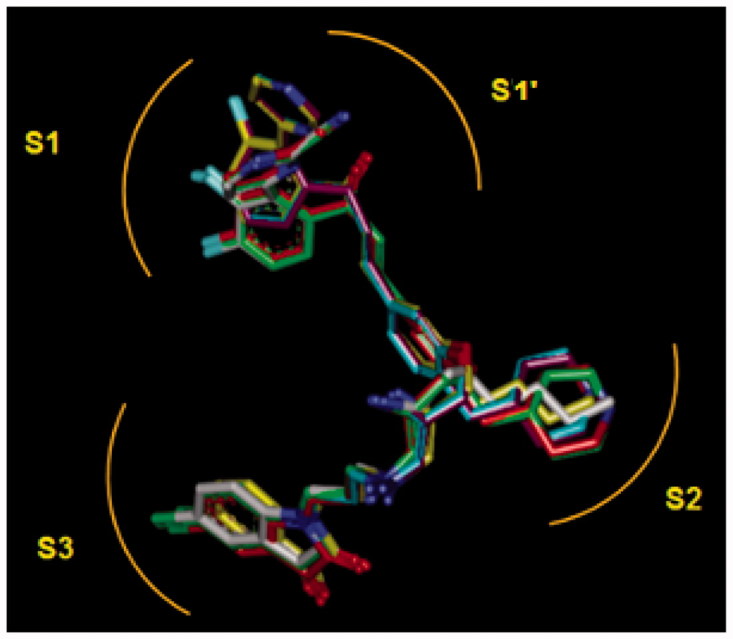
Superimposition of the best analogues exploring the S2 pocket of FP-2 active site; 125–1-1-H-lki-128 (green, *IC*_50_^pre^ = 13 nM), 125–1-1-H-lki-129 (red, *IC*_50_^pre^ = 15 nM), 125–1-1-H-lki-134 (orange, *IC*_50_^pre^ = 18 nM), 127–1-1-H-lki-128 (purple, *IC*_50_^pre^ = 13 nM), 127–1-1-H-lki-129 (blue, *IC*_50_^pre^ = 15 nM), 127–1-1-H-lki-134 (white, *IC*_50_^pre^ = 15 nM).

**Figure 11.**
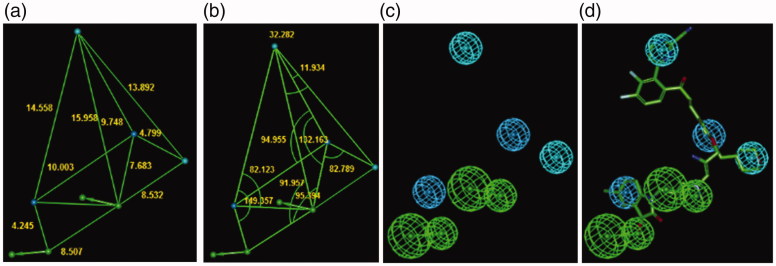
The inhibition pharmacophore filling the S2 pocket of the FP-2 active site derived from the bound conformation of the best analogues with P2 substitution such as 125–1-1-H-lki-128 (*IC*_50_^pre^=13 nM) (green): distances (a), angles (b), features (c), and 125–1-1-H-lki-128 mapping (d). Compared with the 3D QSAR complexation PH4, a supplementary hydrophobic feature corresponding to S2 pocket filling appeared. The features are coloured blue for hydrophobic aliphatic (HYd), green for hydrogen-bond acceptor (HBA), purple for hydrogen-bond donor (HBD) and orange for aromatic (Ar). The arrows represent the projection for the donor and acceptor features.

**Figure 12.**
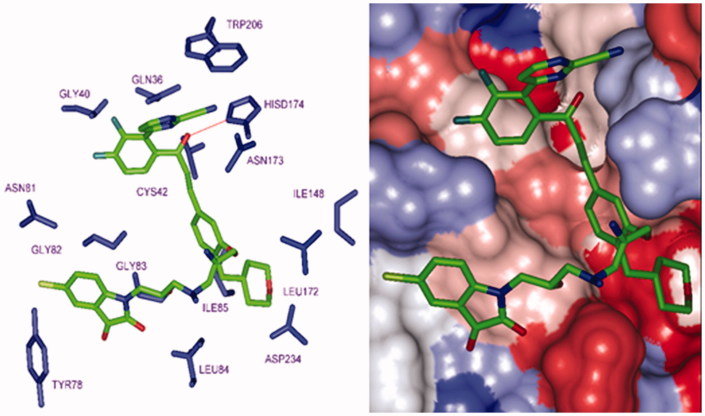
P2 substitution for the S2 pocket filling. (Left): Close up of 125–1-1–H–lki–128 (*IC*_50_^pre^ = 13 nM) at the FP-2 active site. Carbon atoms of interacting residue side chains are coloured blue and those of ligand in green. (Right): Connolly surface of the FP-2 active site for 125–1-1–H–lki–128. The binding site surface is coloured according to residue hydrophobicity: red – hydrophobic, blue – hydrophilic and white – intermediate.

The top scoring virtual hits are HLCIC analogues: 125–1–1–H–lki–H (*IC*_50_^pre^ = 30 nM), 127–1–1–H–lki–H (*IC*_50_^pre^ = 30 nM) without any specific P2 substitution; 125–1–1–H–lki–128 (*IC*_50_^pre^ = 13 nM), 125–1–1–H–lki–129 (*IC*_50_^pre^ = 15 nM), 127–1–1–H–lki–128 (*IC*_50_^pre^ = 13 nM), 127–1–1–H–lki–129 (*IC*_50_^pre^ = 15 nM), 125–1–1–H–lki–137 (*IC*_50_^pre^ = 16 nM), and 127–1–1–H–lki–137 (*IC*_50_^pre^ = 15 nM) with specific substitution targeting S2 pocket filling (*R*_6_ substituent). While the first set displays predicted potency approximately 200 times better than the training set (HLCIC1; Table 10), the last set of new analogues reached *IC*_50_^pre^ 500 times better (Table 11). Despite this exceedingly optimistic picture, our approach helped to identify interesting hydrophobic side chains (*R*_6_-groups) such as (tetrahydro-2H-pyran)Me (128), (piperidin-4-yl)Me (129) and *n*-butane (137) for the S2 pocket filling with a bulkier group compared to the E64 which is decorated only with a short propyl chain at the P2 position. Indeed, from superimposition of the most active training set HLCIC (Figure 9(a)) and the less active ones (Figure 9(b)), we can see that E64 remains the only inhibitor to map the S2 pocket. The superimposition of the best-designed analogues (Figure 10) highlights the significance of hydrophobic contact with the S2 pocket. In fact, an additional pharmacophore derived from these new HLCIC analogues suggests an additional hydrophobic feature located at the S2 pocket (Figure 11). Figure 12 shows the interactions of one of the best-designed analogues 125–1-1–H–lki-128 (*IC*_50_^pre^ = 13 nM) with the FP-2 and the Connolly surface of the binding site shows the lipophilic S2 pocket accommodating a bulkier substituent contributing to better stabilisation and greater affinity. These results are in good agreement with the reported structural information from experimental structure–activity relationship on FP-2 and FP-3 pyrimidine-carbonitrile inhibitors^45^ as well as QSAR model and *in silico* design of dipeptide nitriles inhibitors of FP-3^26^ and FP-2^46^. These conclusions are also in line with the recent SAR study on synthesis and molecular docking of coumarin containing pyrazoline derivatives as promising inhibitors of *in vitro* development of a chloroquine-sensitive (MRC-02) and chloroquine-resistant (RKL-2) strain of *Pf* ^47^.

The ADME-related properties were also computed for the best active designed HLCIC as well as for drug used for the treatment of malaria (Table 12). The chief descriptor, the number of stars (*) deviation of the computed values from the optimum ranges for 95% of known drugs is close to those for known antimalarials. It can be noticed that the human oral absorption in gastrointestinal tract (HOA) is low for the new HLCIC analogues suggesting non-oral delivery. The blood–brain barrier descriptor is in the appropriate range.

**Table 12.** Predicted ADME-related properties of the best designed HLCIC analogues and known antimalarial agents either in clinical use or currently undergoing clinical testing, as computed by QikProp^34^

No	Molecules^a^	#stars^b^	MW [g.mol^−1^]^c^	Smol [A^2^]^d^	Smol, hfo^e^ [Å^2^]	Vmol [Å^3^]^f^	RotB^g^	HBdon^h^	HBacc^i^	logPo/w^j^	logSwat^k^	BIPcaco^l^ [nm.s^−1^]	logB/B^m^	#meta^n^	logKHSA^o^	HOA^p^	%HOA^q^	*IC*_50_^pred^^r^ [nM]
31	125–1–1–H–lki–H	7	686.0	1011	123.2	1899	17	5	16	2.2	−6.1	2.2	−3.6	4	−0.251	1	20.7	30
33	127–1–1–H–lki–H	5	661.0	989	128.4	1834	16	5	15	2.8	−5.3	11.5	−2.7	5	−0.196	1	36.6	30
82	125–1–1–H–lki–128	11	784,2	1135	371,1	2266	19	5	18	4,1	−7,2	7,6	−3,1	6	0,253	1	40.9	13
83	125–1–1–H–lki–129	12	783,2	1178	294,2	2238	19	6	17	2,6	−6,7	0,2	−4,2	5	0,104	1	0	15
84	125–1–1–H–lki–134	12	772.2	1230	303.4	2290	22	6	18	3.0	−8.2	0.3	−5.8	6	0.024	1	0	18
88	127–1–1–H–lki–128	8	759.2	1078	282.8	2099	18	5	17	3.8	−5.8	16.2	−2.6	7	0.068	1	45.1	13
89	127–1–1–H–lki–129	8	758.2	1114	291.2	2130	18	6	16	3.2	−5.1	2.4	−2.6	6	0.098	1	13.7	15
90	127–1–1–H–lki–134	8	747.1	1091	233.0	2096	21	6	17	3.3	−5.4	5.3	−3.5	7	−0.13	1	20.4	17
93	122–1–1–H–lki–130	10	824.3	1190	382.5	2292	18	5	15	5.6	−7.3	7.5	−1.9	6	0.80	1	36.3	26
109	125–1–1–H–lki–132	9	728.2	1082	258.3	2040	18	5	16	3.2	−7.1	3.3	−3.7	5	0.01	1	28.8	29
110	125–1–1–H–lki–133	10	742.2	1128	291.1	2122	19	5	16	3.5	−7.7	2.3	−4.1	5	0.15	1	27.9	20
111	125–1–1–H–lki–137	11	742.2	1141	310.9	2138	20	5	16	3.6	−7.8	2.4	−4.2	5	0.14	1	29.1	16
112	127–1–1–H–lki–132	5	703.1	1041	240.4	1975	17	5	15	3.8	−6.0	19.1	−2.5	6	0.10	1	46.3	23
113	127–1–1–H–lki–133	6	717.2	1072	270.3	2040	18	5	15	4.2	−6.3	19.2	−2.6	6	0.21	1	48.6	19
114	127–1–1–H–lki–137	6	717.2	1080	273.6	2046	19	5	15	4.2	−6.3	17.5	−2.7	6	0.17	1	47.8	15
	Amodiaquine	1	333.7	603	131.7	1019	6	0	5	3.6	−4.8	1689	−0.4	0	0.0	3	100.0	
	Arteether	1	312.4	531	506.0	970	2	0	6	2.7	−3.1	5731.8	0.2	0	−0.2	3	100.0	
	Artemether	1	298.4	491	465.5	902	1	0	6	2.3	−2.9	5729	0.3	0	−0.3	3	100.0	
	Artemisinin	0	282	457	380.6	848	0	0	5	1.7	−2.1	1886	0.1	1	−0.3	3	95.8	
	Artésunate	0	384.4	644	465.1	1156	4	1	8	2.5	−3.9	50.4	−1.4	2	−0.1	3	72.0	
	Dihydroartémisinine	1	284.4	477	395.7	865	1	1	6	1.8	−2.8	1664.9	−0.1	0	−0.1	3	95.4	
	Doxycycline	4	422.3	602	174.0	1104	2	0	17	−4.0*	−0.9	9.2*	−2.5	4	−2.9*	1	20.8	
	Halofantrine	5	470.2	785	160.2	1352	5	0	3	7.6*	−8.5*	2844.1	0.2	0	1.5	1	100.0	
	Luméfantrine	5	496.7	819	160.7	1438	7	0	3	8.3*	−9.4*	4337.2	0.2	0	1.7*	1	100.0	
	Méfloquine	2	362.2	533	0.0	925	2	0	4	4.1	−5.9	2903.1	0.5	0	0.1	3	100.0	
	Tétracycline	5	422.3	605	173.1	1112	2	0	16	−3.4*	−1.4	6.8*	−2.6	5	−2.5*	1	21.8*	

^a^Designed analogues (Table 10).

^b^Drug likeness, number of property descriptors (from 24 out of the full list of 49 descriptors of QikProp, version 3.7, release 14) that fall outside of the range of values for 95% of known drugs.

^c^Molecular weight in g.mol^−1^ (range for 95% of drugs: 130–725 g.mol^−1^).

^d^Total solvent-accessible molecular surface, in Å^2^ (probe radius 1.4 Å) (range for 95% of drugs: 300–1000 Å^2^).

^e^Hydrophobic portion of the solvent-accessible molecular surface, in Å^2^ (probe radius 1.4 Å) (range for 95% of drugs: 0–750 Å^2^).

^f^Total volume of molecule enclosed by solvent-accessible molecular surface, in Å^3^ (probe radius 1.4 Å) (range for 95% of drugs: 500–2000 Å^3^).

^g^Number of non-trivial (not CX3), non-hindered (not alkene, amide, small ring) rotatable bonds (range for 95% of drugs: 0–15).

^h^Estimated number of hydrogen bonds that could be donated by the solute to water molecules in solution, averaged over a number of configurations (range for 95% of drugs: 0.0–6.0).

^i^Estimated number of hydrogen bonds that could be accepted by the solute from water molecules, averaged taken over a number of configurations (range for 95% of drugs: 2.0–20.0).

^j^Logarithm of partitioning coefficient between *n*-octanol and water phases (range for 95% of drugs: −2 to 6.5).

^k^Logarithm of predicted aqueous solubility, log S. S in mol⋅dm^−3^ is the concentration of the solute in a saturated solution that is in equilibrium with the crystalline solid (range for 95% of drugs: −6.0 to 0.5).

^l^Predicted apparent Caco-2 cell membrane permeability in Boehringer-Ingelheim scale, in [nm⋅s ^−1^] (range for 95% of drugs: <25 poor, >500 great).

^m^Logarithm of predicted brain/blood partition coefficient. (range for 95% of drugs: −3.0 to 1.2).

^n^Number of likely metabolic reactions (range for 95% of drugs: 1–8).

^o^Logarithm of predicted binding constant to human serum albumin (range for 95% of drugs: −1.5 to 1.5).

^p^Human oral absorption (1: low; 2: medium; 3: high).

^q^Percentage of human oral absorption in gastrointestinal tract (<25% poor, >80% high).

^r^Predicted inhibition constants *IC*_50_^pre^. *IC*_50_^pre^ was predicted from computed ΔΔ*G*_com_ using the regression equation shown in Table 3.

*Asterisk indicates that the property descriptor value falls outside the range of values for 95% of known drugs.

## 4. Conclusion

Natural-product-like hybrids design for *pf*FP-2 inhibition stems from the multiple need to provide naturally occurring, resistance overcoming, and favourable pharmacokinetic profile compounds mimicking in this way, how nature synthesise by exploring efficiently chemical space. HLCIC antimalarials structural requirement for falcipain 2 inhibition has been assessed from the complexation “one descriptor” QSAR correlating GFE upon *pf*FP-2:HLCIC complex formation with activity. Moreover the derived 3D QSAR Pharmacophore was augmented to S2 pocket filling in order to provide a complete PH4 able to guide synthesis of novel potent isatin–chalcone inhibitors. Virtual screening of large and diverse VL of HLCIC analogues by the PH4 led to identification of 125–1–1–H–lki–H (30 nM), 127–1–1–H–lki–H (30 nM) 125–1–1–H–lki–128 (13 nM), 125–1–1–H–lki–129 (15 nM), 127–1–1–H–lki–128 (13 nM), 127–1–1–H–lki–129 (15 nM), 125–1–1–H–lki–137 (16 nM) and 127–1–1–H–lki–137 (15 nM); the best ones according to our design strategy based on S2 hydrophobic contact display predicted potency reaching 200 times that of the most active training set HLCIC1. They are recommended for synthesis and evaluation to check the efficiency of our design approach and guide future discovery of non-peptide, natural product like hybrids FP-2 inhibitors.
